# Individual-based predominance of visual input in multisensorial integration for balance is correlated with proprioceptive drift in rubber hand illusion

**DOI:** 10.1038/s41598-023-39253-9

**Published:** 2023-07-24

**Authors:** Esra Özkan, Ceyda Özler, Kardelen Akar, Hussein Youssef, Kaan Özmen, Zümrüt Duygu Şen, Atay Vural, Yasemin Gürsoy-Özdemir

**Affiliations:** 1 Koç University Research Center for Translational Medicine, Koç University Hospital, Zeytinburnu, 34010 Istanbul, Turkey; 2 Department of Neurology, School of Medicine, Koç University, Istanbul, Turkey; 3 Department of Psychiatry and Psychotherapy, Jena University Hospital, Jena, Germany; 4 Center for Intervention and Research on Adaptive and Maladaptive Brain Circuits Underlying Mental Health, DZP, Jena, Germany; 5 Clinical Affective Neuroimaging Laboratory (CANLAB), Magdeburg, Germany; 6 Department of Psychiatry and Psychotherapy, University Tübingen, Tübingen, Germany

**Keywords:** Sensory processing, Human behaviour, Perception

## Abstract

Rubber hand illusion (RHI) is a traditional task that examines multisensory integration. The visual capture of tactile stimulus given to the seen rubber hand was considered to predominate the sensory processing and interfere with the bottom-up proprioceptive and tactile inputs received from the unseen real hand that results in mislocalization of participants hand towards rubber hand, namely proprioceptive drift (PD). Another task that requires multisensorial integration and shows a predominance of visual input is the maintenance of body posture. However, if the predominance of visual input in one task is generalizable to another task is yet to be elucidated. We aimed to examine if individual dependency on visual inputs in multisensorial integration in balance correlated with PD in RHI. Twenty healthy participants were recruited for the study and completed the RHI task. The contribution of visual inputs to the static body balance was measured with the instrumented clinical test of sensory interaction for balance and indexed with Romberg Quotient (RQ). We found a moderate positive correlation between PD and RQ. Individuals with more dependence on visual information in maintaining body posture had higher PD in RHI. Our results indicate that there can be an individual-based dependence on particular domains of sensory input preserved during different tasks of multisensorial integration. Future studies must clarify whether this tendency relates to certain physical or physiological traits.

## Introduction

Multisensory integration refers to the way in which the processing of stimuli from one sensory modality is sensitive to the information provided by stimuli from another. Information coming from several modalities can thus be integrated to improve perception and solve cross-modal conflicts^1^. The multisensory integration of body-related stimuli has been recently investigated with bodily illusions induced by experimental protocols, such as the rubber hand illusion^2^.

Rubber hand illusion (RHI) is a well-known phenomenon used to study the sensation of body ownership and proprioceptive localization of a body part^2^. In the visual RHI paradigm, a rubber hand is placed within the visual field near the participant's hidden real hand, and synchronous tactile stimulation is given simultaneously to the rubber hand and the real hand. This induces an illusory sense of ownership over the rubber hand in addition to a mislocalization of the real hand, namely proprioceptive drift (PD), where the participant's perceived hand position shifts towards the rubber hand. RHI is considered to be a result of the multisensorial integration of visual, tactile, and proprioceptive inputs, along with a complex top-down influence of stored body representations^3–6^. PD is thought to reflect the brain's updating of its body representation to incorporate the rubber hand as part of the body schema. However, the subjective embodiment (body ownership) scores and the extent of proprioceptive drift do not always go parallel^7,8^. Therefore, further examination of the mechanisms underlying PD is needed.

The localization of body parts is a complex process that involves the integration of sensory information from multiple sources, including proprioception, vision, touch, and audition. Proprioception is the sense of the body's position and movement in space, which is mediated by the specialized receptors located in the muscles and tendons. In RHI, the visuotactile signals interfere with the proprioceptive inputs. The brain integrates this information based on the spatiotemporal properties of the visual, tactile, and proprioceptive cues. Several studies investigated the impact of various spatiotemporal properties on RHI; for example, PD is shown to be positively correlated with a longer duration of synchronous tactile stroking^7,9^ and shorter distance between the real and rubber hands^10^, suggesting the stronger congruency between sensory modalities increases PD. On the other hand, slow displacement of the real hand decreases PD, probably because of increasing the proprioceptive data gathered from the participant’s real hand^11^. Additionally, if the distance is maintained, the incongruent positioning of the rubber hand to the real hand does not affect the amount of drift^12^. Moreover, asynchronous stroking could result in PD if it is not continuously maintained^8^ or even after continuous asynchronous stroking, a smaller yet significant PD was reported^13^. Thus, it can be argued that asynchronous stimulation diminishes the proprioceptive drift after tactile mismatch and can be able to overcome visual illusion^7^.

Besides the spatiotemporal properties of stimulation, the individual sensitivity of subjects to specific signals in multisensorial integration may affect RHI. One of the parameters studied was the participant’s proprioceptive acuity, which can be expected to be reversely correlated with PD. Two studies from the same group reported that neither illusion nor the embodiment scores were affected by the subject’s proprioceptive acuity^14,15^. However, another study showed a weak reverse correlation between the strength of proprioception and the ownership of the rubber hand but not with the reported position of the hand^16^.

Moreover, interoceptive sensitivity regarding one’s awareness of their heartbeats was found to be predictive of PD and embodiment of the fake hand^17^, whereas it could not be replicated^16,18^. Interestingly, when their interoceptive signals were given to the participants synchronously as visual cues like flashing lights of the virtual body or hand during illusion, referred to as ‘online,’ it strengthened the RHI^19,20^. So, the visual capture of interoceptive signals, or tactile signals as explained above, weaken the bottom-up proprioceptive inputs and lead the felt localization of the hand to move towards the visual-captured position. Moreover, another study reported that if the participants were asked to focus on the visual signals, the illusion also strengthened^21^. Thus, reweighting visual cues with or without^8^ combined with other stimuli is particularly important in PD. Therefore, an examination of the association between PD in RHI and the dependence on visual signals during another multi-sensorial integration task would shed light on the underlying mechanisms.

Maintaining the body balance requires multisensory integration as the RHI. Various inputs of somatosensory, visual, and vestibular systems are continuously accumulating^22^; nevertheless, the sensory input is dynamically reweighted if one domain is blocked (i.e., eyes closed)^22^. The information from the somatosensory system is internal and about the position of one’s own body. On the other hand, the visual and vestibular systems provide an external source of information about the body's position with respect to the surroundings^22,23^. The somatosensory system depends on proprioceptive information crucial to the standing balance, and studies estimate its contribution as 58–69%, depending on the condition^24^.

The clinical test of sensory interaction for balance (CTSIB) is used to assess sensory integration for balance in a quiet standing position with eyes open or closed either on a firm or foam surface^25^. CTSIB is validated with inertial opal sensors for accurate and sensitive measurements^26^. The total sway area is the well-established indicator of the standing balance, and it has been shown to increase with somatosensory, visual, and vestibular deprivation conditions separately^22,26–28^. Moreover, total sway area was proven to be sensitive in detecting aging-associated changes of balance^29^, as well as changes in diseases like multiple sclerosis (MS)^30^ and Parkinson's disease^26,31^.

In this study, we hypothesized that individual-based dependence on visual input in multi-sensorial integration of balance, the individual’s “off-line” (not temporally related to RHI) tendency to rely on visual inputs, is associated with the proprioceptive drift in RHI, the extent of reliance on the visual capture of the illusion inducing stimuli (tactile, interoceptive, etc.). Our hypothesis is founded on the assumption that individuals may exhibit a predisposition to prioritize certain sensory information over others during the process of multisensory integration. This personal inclination, or "trait," could persist across various tasks. While previous investigations have examined different factors relating to individual tendencies in the RHI, such as interoceptive sensitivity, proprioceptive acuity, or hypnotizability, a consensus has yet to be reached, making further elucidation necessary. In this preliminary study, we have selected standing balance and the RHI as distinct multisensory integration tasks in order to explore any potential correlation between individuals' reliance on visual inputs in these tasks. The contribution of visual input to the balance was calculated as the postural sway’s ratio in only visual deprivation condition to all domains available condition in CTSIB, formerly named as Romberg Quotient (RQ) in studies^25,30^. On the other hand, the magnitude of PD in the RHI task has been acknowledged as an indicator of visual dominance. This conclusion is supported by a substantial body of evidence mentioned earlier, which suggests that PD strengthens through the reevaluation of visual information over other sources. We assumed that RQ might show a positive correlation with PD.

## Methods

### Ethics

The study was approved by Koç University Ethical Committee, granted with the decision number 2020.418.IRB1.157. All participants signed written informed consent before commencing the study. This study was conducted in line with the declaration of Helsinki.

### Study design and participants

The study was conducted in the motion analysis lab at Koç University Hospital (KUTTAM) between December 2021 and March 2022. Twenty healthy participants (14 females and six males) were recruited. They have been included in the study if they are healthy, aged ≥ 18 years, with no previously reported neurological or orthopedic conditions that could affect their body balance, and if they confirmed no previous participation in any rubber hand illusion experiment. Exclusion criteria included participants with a history of neurological, psychiatric, somatosensory conditions, or cognitive dysfunction. Each participant reported their handedness; one out of 20 participants was left-handed, and the rest were right-handed.

### Rubber hand illusion

**Figure 1:**
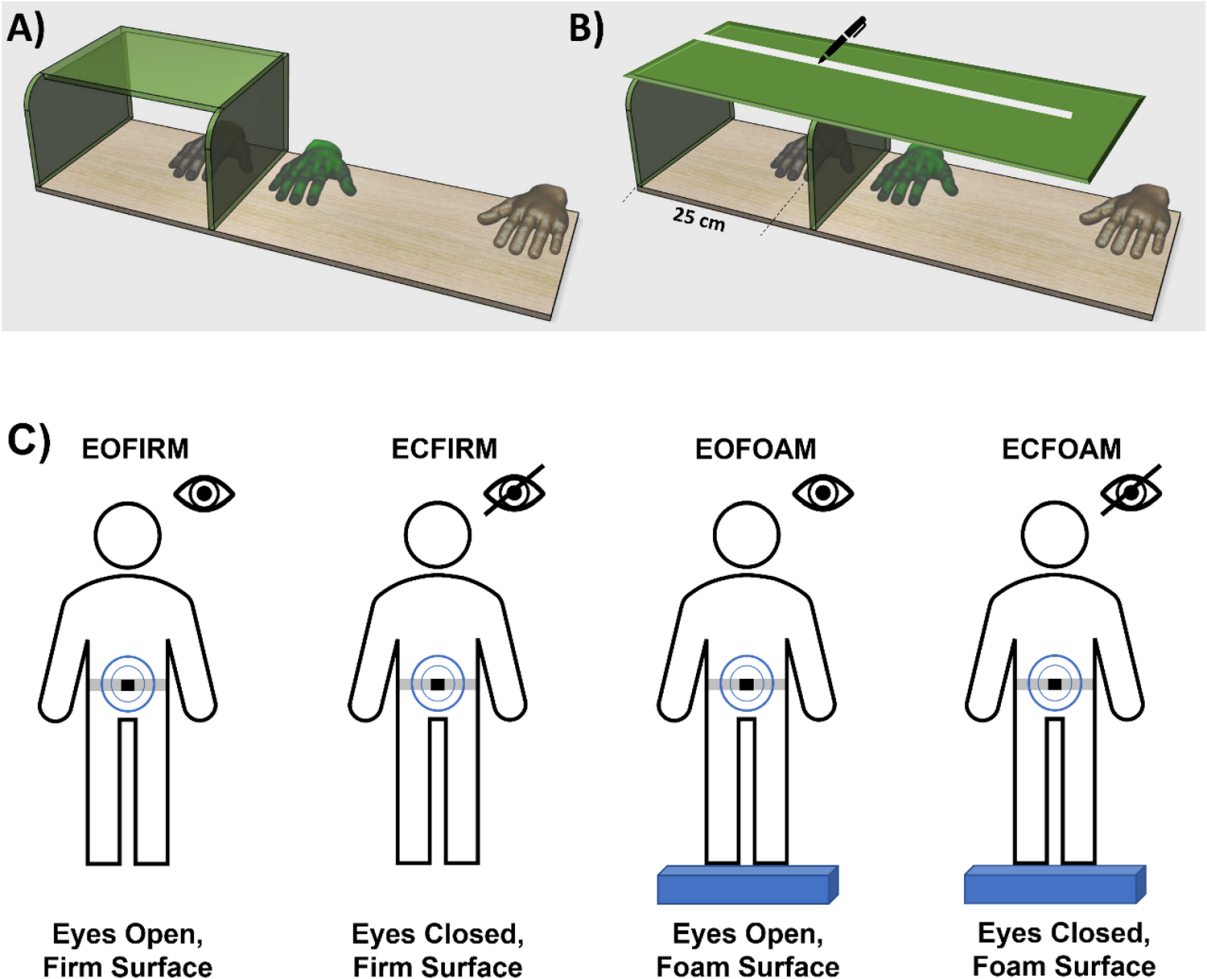
The schematic presentation of RHI and mCTSIB. (**A**) The RHI setup is shown. The participants’ real left hand is placed on a table and is not visible to them. A rubber hand (here green), visible to the participant, is placed outside the box. (**B**) For measuring proprioceptive drift, a panel covering both real hand and rubber hand is placed, and the participant is asked to mark the place of their left index finger. (**C**) Four conditions of mCTSIB were shown. The localization of the initial wireless sensor was placed on the fifth vertebrae.

Each participant was asked to wear a laboratory coat and sit comfortably in front of a table. The environment was isolated from the lab by panels surrounding the researcher and the participant, and the researcher sat facing the participant. A rectangular box on the table on the left side with two openings at each side confronting both the participant and the researcher. The participant’s left hand was gently placed with the assistance of the researcher—covered within the box—and the participant’s left index finger was placed at the same predefined point. Next to the box, a rubber hand -resembling the left hand- was attached to the same color laboratory coat worn by each participant to enhance the impression that the rubber hand was their real hand. Afterward, another coat is set to cover the participant's left shoulder, and opening of the box at their side, and the ending of the rubber hand. The investigator briefly explained the procedures to each participant using the same language and words in a smooth manner. The researcher strokes the rubber hand and the participant’s own hand's index finger with two brushes for 120 s, either synchronously or asynchronously, at 1 Hz. Each participant received three synchronous and three asynchronous sessions, and after all sessions, a large board was placed covering the box and the rubber hand. Next to each participant, a plain paper strip on the board, and the participants were asked to mark the position where they indicated their left index finger. Each time a new strip was placed on the panel so that the old mark was not visible to the participant, Fig. 1A,B.

### CTSIB

The contribution of visual, proprioceptive, and vestibular inputs to the balance in the quiet standing condition was assessed with CTSIB^25^. The current study evaluated modified CTSIB (mCTSIB) using motion analysis-based sensors for objectively and accurately tracking balance parameters^26^. mCTSIB is a modified version of CTSIB, developed to use only four out of six conditions that were used to be included in CTSIB^32^. Balance analysis was performed using a wireless inertial sensor system (Opals and Mobility Lab, APDM Inc., USA). Wearable sensors were attached bilaterally to feet by an elastic strap and a third sensor at the fifth lumber vertebrae. mCTSIB test is composed of four interconnected tests challenging the participant’s balance through different conditions. Each participant stands barefoot, socks on, and separates feet by feet template to standardize foot placement for all participants, as summarized in Fig. 1C. The data were automatically collected and analyzed in MobilityLab^33^. For detecting the individual-based contributions of vision to the static balance, the Romberg quotient was used as reported by previous studies^25,30^, which is the ratio of total sway area in firm surface-eyes closed condition to firm surface-eyes open condition.

### Data analysis

Data were analyzed using SPSS 26.0 software for Windows (IBM, New York, USA), and GraphPad Prism software 8.4.3 (GraphPad Software Inc., La Jolla, CA, USA) was used for figure generation. Since we are exploring a novel correlation between PD and RQ, we included 10 participants for a pilot study and did a correlation analysis between PD and RQ. We found a strong positive correlation between the two parameters (r = 0.81, p = 0.005, Spearman rank order correlation test). We calculated the estimated sample size based on this correlation value, to show significance with 0.90 power and an alpha value of 0.05 as a minimum of 15 participants (SPSS, Power analysis for correlation, Spearman rank-order correlation). Since the most relevant study with our hypothesis in the literature included 28 participants^34^, we decided our sample size to be 20 and recruited 10 more participants after the pilot study. Normality assumptions were inspected with the Shapiro–Wilk test. Continuous variables were presented as median (interquartile range [IQR]), and categorical variables were presented as percentages. The participant showed 1 cm or above proprioceptive drift towards the rubber hand in any of three synchronous stroking sessions assigned to the RHI (+) group^8^. The remaining participants whose PD towards the rubber hand was smaller than 1 cm for all synchronous stroking sessions were assigned to the RHI (−) group. Proprioceptive drift values correspond to the mathematical average of the measurements done at the three synchronous or asynchronous stroking sessions. Simple chi-square and Fisher exact tests were used to compare categorical variables between groups defined by RHI presence. The Mann–Whitney-U test was used to evaluate the distribution of the Romberg quotient and PD between these groups and the Spearman test for their correlation. The statistical significance of all the tests was applied two-tailed and accepted at p < 0.05.

## Results

### Demographics and characteristics of participants

The median age of the participants was 26.0 (24.3–27.0). Fourteen (70%) were female, and six (30%) were male participants. The distribution of age did not differ according to sex (p = 0.841, Mann–Whitney *U* test). Nineteen (95%) out of 20 participants were right-handed. In the current study, 75% (15/20) of participants experienced the rubber hand illusion in line with the literature^2^. The demographics of the participants are illustrated in Table 1. Age (p > 0.05, Mann Whitney *U* test), sex, and handedness were similar between groups (p > 0.05 for both, Fisher’s exact test). The PD was significantly more prominent in the RHI (+) group, as expected (p < 0.001, Mann Whitney *U* test). Also, the Romberg Quotient was significantly higher in those who experienced RHI (p = 0.042, Mann–Whitney *U* test).

**Table 1:** Descriptive statistics of the participants according to the presence of the RHI phenomenon.

Variable	Total (*n* = 20)	RHI (+) (*n* = 15)	RHI (−) (*n* = 5)	P-value
Female sex (%)	70.0	80.0	40.0	0.131
Age (years)	26.0 (24.3–27.0)	26.0 (25.0–28.0)	25.0 (23.0–26.5)	0.349
Right handedness (%)	95.0	93.3	100.0	1.000
Proprioceptive drift (cm)*	1.6 (0.0–3.2)	3.0 (1.2–3.7)	− 0.7 (− 3.0 to 0.1)	** < 0.001**
Sway area, EOFIRM (m^2^/s^4^)	0.02 (0.01–0.04)	0.02 (0.01–0.04)	0.03 (0.01–0.06)	0.612
Sway area, ECFIRM (m^2^/s^4^)	0.02 (0.01–0.04)	0.02 (0.01–0.04)	0.02 (0.01–0.04)	0.612
Sway area, EOFOAM (m^2^/s^4^)	0.06 (0.05–0.09)	0.06 (0.05–0.10)	0.07 (0.03–0.09)	0.735
Sway area, ECFOAM (m^2^/s^4^)	0.13 (0.09–0.16)	0.13 (0.11–0.15)	0.11 (0.08–0.18)	0.735
Romberg quotient	0.9 (0.7–1.4)	1.2 (0.8–1.5)	0.6 (0.5–1.0)	**0.042**

Data are presented as median (IQR) or as percentages. Statistically significant p-values showed in bold.

*Shows the average proprioceptive drift measured after the synchronous stroking sessions.

*ECFIRM* eyes closed/firm surface, *ECFOAM* eyes closed/foam surface, *EOFIRM* eyes open/firm surface, *EOFOAM* eyes open/foam surface, *RHI* rubber hand illusion, *RHI (*+*)* PD ≥ 1 cm for at least one synchronous session, *RHI (−)* PD < 1 cm for all synchronous sessions.

### Rubber hand illusion and associated proprioceptive drift

**Figure 2:**
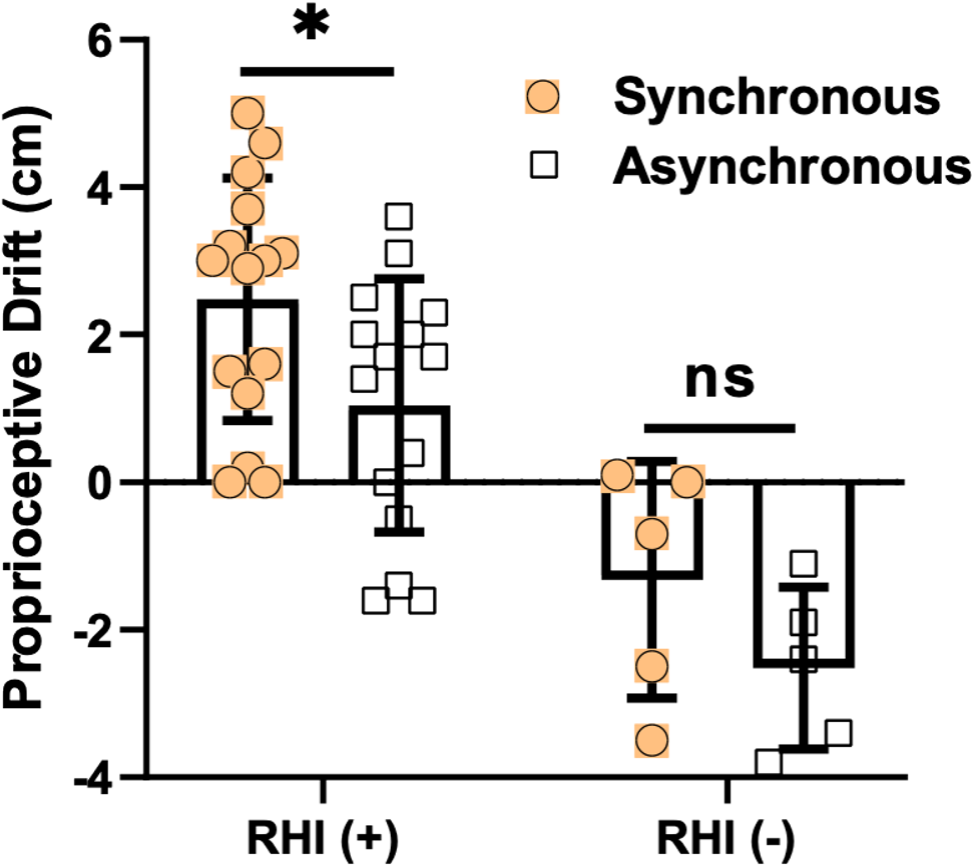
Rubber hand illusion parameters. The proprioceptive drift was significantly larger after synchronous than asynchronous stroking in the RHI (+) group (p = 0.047) but not in the RHI (−) group. *RHI* rubber hand illusion, *RHI (*+*)* PD ≥ 1 cm for at least one synchronous session, *RHI (−)* PD < 1 cm for all synchronous sessions.

Proprioceptive drift was 1.6 (0.0–3.2) cm for synchronous stroking sessions and 0.2 (− 1.6 to 2.0) cm for asynchronous stroking sessions in the study group. The difference was not statistically significant (p = 0.075, Mann–Whitney *U* test). When the groups were analyzed separately, the proprioceptive drift was significantly larger after synchronous than asynchronous stroking in the RHI (+) group (p = 0.046, Mann–Whitney *U* test) but not in the RHI (−) group (p = 0.310, Mann–Whitney *U* test), Table 1, Fig. 2.

### Association between RHI and mCTSIB

**Figure 3:**
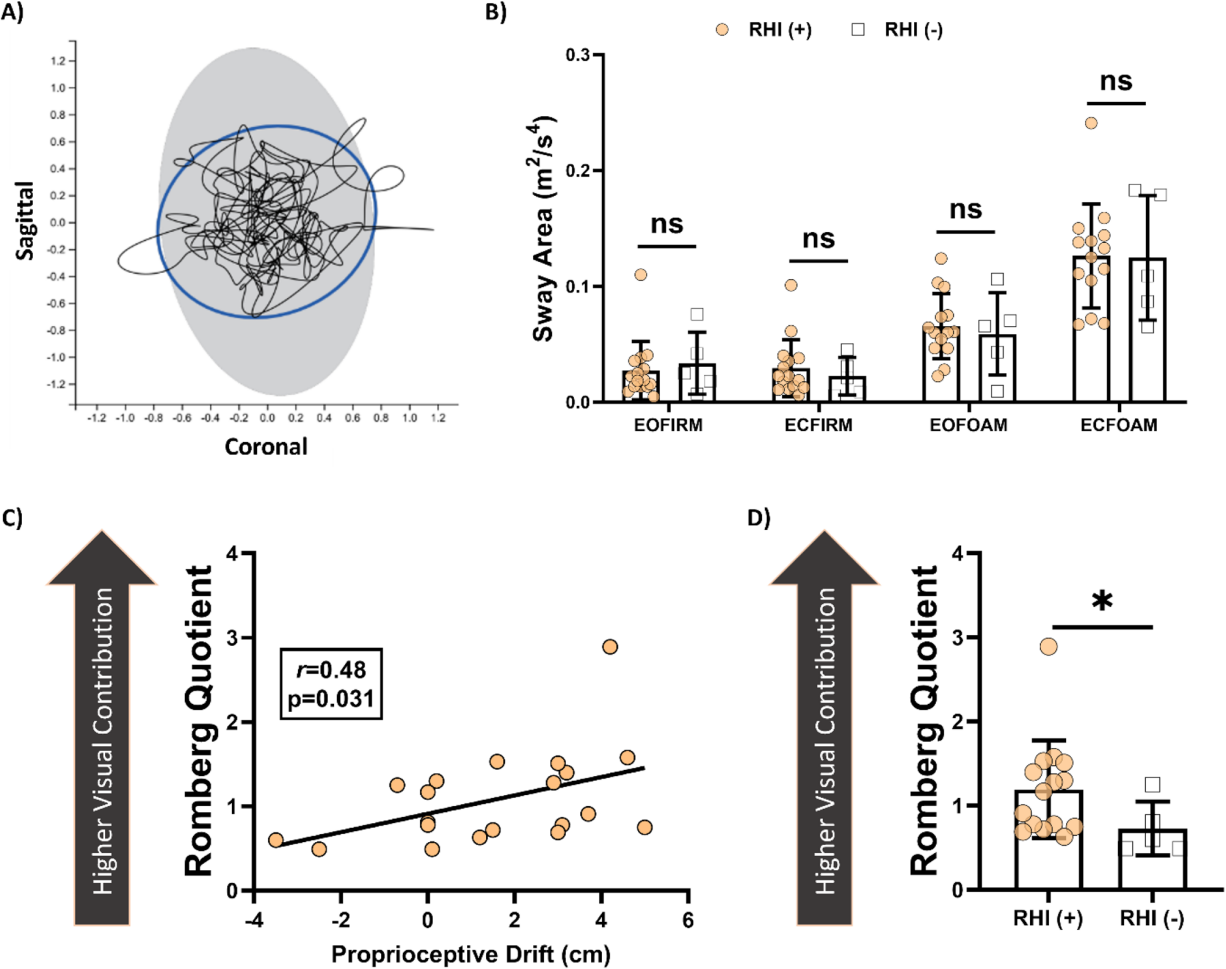
CTSIB parameters. (**A**) A representative image of postural sway, scribbles representing the body sway of the participant in the coronal plane, and the gray area representing the average normal sway area in the population. (**B**) The total sway area was similar between groups in all four CTSIB conditions. (**C**) The Romberg quotient was positively correlated with proprioceptive drift. (**D**) The ratio of Sway area at ECfirm to EOfirm conditions was calculated (Romberg quotient) and found to be significantly higher in the RHI (+) group (p = 0.042). *ECfirm* eyes closed/firm surface, *EOfirm* eyes open/firm surface, *EOfoam* eyes open/foam surface, *RHI* rubber hand illusion, *RHI (*+*)* PD ≥ 1 cm for at least one synchronous session, *RHI (−)* PD < 1 cm for all synchronous sessions.

The total sway area was obtained from four distinguished conditions; eyes open/closed on a firm surface and eyes open/closed on a foam surface, abbreviated EOfirm, ECfirm, EOfoam, and ECfoam, respectively. For all participants, the total sway area was in the normal range identified by the software according to the population studies at all four conditions^26–28^, Fig. 3A. Hence, all participants in our study have a normal static body balance.

Moreover, for exploratory reasons, we checked the distribution of total sway area between study groups defined by the presence of the RHI, either RHI (+) or RHI (−). The total sway area did not significantly differ between different mCTSIB test conditions among groups (p > 0.05 for all, Mann–Whitney *U* test), as shown in Fig. 3B.

We calculated the Romberg quotient, which represents the individual-based contribution of vision to the standing balance. In line with our hypothesis, RQ showed a moderate correlation with PD (r = 0.47, p = 0.035, Spearman’s rank order correlation), Fig. 3C. Furthermore, RQ was significantly higher in the RHI (+) group (p = 0.042), Fig. 3D. Thus, the higher dependence on the visual input for maintaining the body posture was associated with higher proprioceptive drift.

## Discussion

Rubber hand illusion is a simple and widely applied paradigm to study the bodily self and multisensorial integration. In our study, young and healthy participants completed the RHI task. Those who reported their hand’s localization towards rubber hand accounted for 75% of the total, consistent with the previous reports^2^. In line with our hypothesis, PD was positively correlated with the RQ, which indexes the contribution of visual information on the maintenance of body posture. This result might suggest that the degree of dominance of visual input in multisensory integration can be a general characteristic of the individuals that are applied in processing different sensory inputs.

The PD was significantly bigger after synchronous stroking sessions than asynchronous ones in the RHI (+) group. But also asynchronous stroking resulted in PD towards the rubber hand in the same group. This is consistent with the literature that PD can occur with asynchronous stroking or even simply observing^8,13^. On the other hand, we did not observe a significant difference in PD between synchronous and asynchronous stroking in the RHI (−) group. Additionally, we noticed a tendency for the hand to be mislocalized away from the rubber hand. This occurrence could be attributed to the limited number of participants assigned to the RHI (−) group, but further studies are necessary. Including an embodiment questionnaire would help eliminate the possibility that these results are a result of poor compliance from those participants with the task.

The CTSIB was shown to be sensitive enough to detect changes in visual, somatosensory, and vestibular deprivation conditions^22,26–28^, with age^29^, or in certain diseases like MS^30^ and Parkinson's disease^26,31^, and even in between the participants doing a counting down task and those did not^35^. On the other hand, PD is thought to stem from the congruency of the visual and tactile stimulations that interfere with the intrinsic proprioceptive information collected from the real hand. Several studies manipulated the spatiotemporal properties of visuotactile manipulations and supported this idea^7–12^. One may argue the comparability of two multisensory integration tasks, RHI and static body balance, since the latter includes the integration of vestibular input, which is traditionally not considered effective in the former. But Ponzo et al. showed that vestibular stimulation during RHI increases PD, thus strengthening the effect of visual capture over proprioception, suggesting the role of vestibular function in the multisensorial integration of RHI^36^. Moreover, our findings might indicate a more general predominance of visual information in multisensory integration in certain individuals.

The effect of individual-based differences in proprioception^14–16^ and interoception^16–20^, as well as psychological traits like hypnotizability^37^ to RHI, were questioned by several studies with inconsistent results. These studies were designed to search for a relationship between the individual’s performance on a particular sensory domain, like the ability to be aware of their own heartbeats or accuracy in two-point discrimination. But if the individual’s dependence on a sensory domain over others during multisensorial integration, like visual over other domains in this study, correlated with the same tendency in another multisensorial integration task, has not been studied before.

One interesting study demonstrated that old adults with a lower risk of falling assessed with the instrumented Timed Up and Go (TUG) test had weaker illusion scores in the synchronous condition of the rubber foot illusion (RFI) than the old adults with a high risk of falling and young adults^34^. The study also includes measurements of the visual accuracy and tactile sensitivity of the participants, and they were reported to be similar between young and old adults. The researchers discuss this relationship between a balance parameter and body representation as the low-risk group may cease to rely on visual inputs that may be degraded and untrustable in older age. Thus, older adults with a low risk of falling in that study may correspond to the participants with a lower contribution of vision to the balance in our study. Our findings support the explanation of the researchers, not the visual accuracy but dependence on vision may interfere with illusion. Moreover, another study reported that if the participants were asked to focus on visual inputs during the RHI experiment, the embodiment of the rubber hand strengths. The same study also showed that when the task instruction given is “focus on tactile signals from the real hand,” the ownership of the fake hand was somewhat mitigated. These results align with our study that not only the individual tendency to rely on vision but experimentally reweighting the visual signals by an attention task also promotes illusion^21^.

Functional magnetic resonance imaging (fMRI) studies pointed out that multisensorial integration in RHI is associated with the cerebellum and parietal, insular, premotor, and frontal opercular regions of the cerebral cortex^38^. Sensory integration of balance is also simulated in fMRI, and activated areas reported as middle and superior temporal gyri, insula, and a large cluster that covered the corpus callosum, superior and medial frontal gyri, as well as the anterior cingulate and caudate nucleus^39^. Although shared areas are activated between two tasks, insular and frontal regions, further experiments were needed to clarify neuroanatomical correlates underpinning the shared tendency to rely on visual inputs.

The current study has several limitations. First, the sample size of the study is small. But, although a small number of participants were recruited, a homogenous cohort of participants was achieved, and with this sample size, statistically meaningful findings were obtained that is comparable with previous studies. Moreover, 25% of our study group (only 5 participants) were in the RHI (−) group, which may interfere with the strength of the statistical analysis since observed group differences would be very sensitive to individual variations. Thus, we emphasized mostly the correlation between PD and RQ rather than group comparisons.

Another limitation of the study is that we did not include an embodiment questionnaire, so we could not discuss the effect of individual vision dependency on the sense of ownership of the fake hand. Further studies are needed to show if the embodiment of the rubber hand is related to RQ, as demonstrated for PD in this study.

## Conclusion

Our study showed that relying on visual inputs to maintain body balance correlates with PD in RHI. Individuals with more dependence on visual information have higher PD. This may suggest that there can be an individual-based predominance of certain sensorial input domains during different tasks requiring multisensorial integration. Future studies are needed to clarify our results’ generalizability to the sense of body ownership and their relevance in certain physical and physiological traits or diseases.

## Data Availability

The data supporting this study’s findings are available from the corresponding author, EÖ, upon request.
